# Impact of altered glycaemia on blood-brain barrier endothelium: an *in vitro* study using the hCMEC/D3 cell line

**DOI:** 10.1186/2045-8118-11-8

**Published:** 2014-04-05

**Authors:** Ravi K Sajja, Shikha Prasad, Luca Cucullo

**Affiliations:** 1 Department of Pharmaceutical Sciences, School of Pharmacy, Texas Tech University Health Sciences Center, 1300 S. Coulter Street, Amarillo, TX 79106, USA; 2 Center for Blood Brain Barrier Research, Texas Tech University Health Sciences Center, Amarillo, TX 79106, USA

**Keywords:** Diabetes, hCMEC/D3 cell line, Glucose transporter, Oxidative stress, Permeability, Tight junctions, Blood-brain barrier, Inflammation, Cytokine, Alternative

## Abstract

**Background:**

Cerebrovascular complications involving endothelial dysfunction at the blood-brain barrier (BBB) are central to the pathogenesis of diabetes-related CNS disorders. However, clinical and experimental studies have reported contrasting evidence in relation to the effects of hyperglycemia on BBB permeability and function. Similarly the effect of hypoglycemia on BBB integrity is not well understood. Therefore, we assessed the differential impact of hypo and hyperglycemic conditions on BBB integrity and endothelial function *in vitro* using hCMEC/D3, a well characterized human brain microvascular endothelial cell line.

**Methods:**

Parallel monolayers of hCMEC/D3 were exposed to normal, hypo- or hyperglycemic media, containing 5.5, 2.2 or 35 mM D-glucose, respectively. Following 3-24h exposure, the expression and distribution of BBB tight junction (ZO-1 and claudin-5) adherence junction (VE-cadherin) proteins, and glucose transporters as well as inflammatory (VCAM-1) and oxidative stress (Nrf-2) markers were analyzed by immunofluorescence and western blotting. Endothelial release of growth factors and pro-inflammatory cytokines were determined by ELISA. Further, the impact of altered glycemia on BBB permeability was assessed in hCMEC/D3 – astrocyte co-cultures on Transwell supports using fluorescent dextrans (4–70 kDa).

**Results:**

Compared to controls, exposure to hypoglycemia (3 and 24h) down-regulated the expression of claudin-5 and disrupted the ZO-1 localization at cell-cell contacts, while hyperglycemia marginally reduced claudin-5 expression without affecting ZO-1 distribution. Permeability to dextrans (4-10 kDa) and VEGF release at 24h were significantly increased by hypo- and hyperglycemia, although 70 kDa dextran permeability was increased only under hypoglycemic conditions. The expression of SGLT-1 was up-regulated at 24h hypoglycemic exposure while only a modest increase of GLUT-1 expression was observed. In addition, the expression of Nrf-2 and release of interleukin-6 and PDGF-BB, were down-regulated by hypoglycemia (but not hyperglycemia), while both conditions induced a marginal and transient increase in VCAM-1 expression from 3 to 24h, including a significant increase in VE-cadherin expression at 3 h following hyperglycemia.

**Conclusions:**

In summary, our findings demonstrate a potential impairment of BBB integrity and function by hypo or hyperglycemia, through altered expression/distribution of TJ proteins and nutrient transporters. In addition, hypoglycemic exposure severely affects the expression of oxidative and inflammatory stress markers of BBB endothelium.

## Introduction

The blood-brain barrier (BBB), a dynamic interface between systemic circulation and brain, precisely regulates the CNS microenvironment [1]. The BBB selectively restricts blood-borne and xenobiotic entities from entering the CNS, thus maintaining cerebral homeostasis. These restrictive properties are bestowed upon the BBB by unique features of the microcapillary endothelium such as: 1) expression and organization of intercellular tight junction (TJ) complexes [2,3]; 2) polarized expression of specialized carrier systems for selective transport of essential nutrients; 3) non-selective drug efflux pumps [4,5]. Thus, BBB integrity is critical to ensure optimal CNS function and its disruption during cerebrovascular pathologies can be prodromal to the onset and progression of major neurological disorders [6].

Increasing evidence indicts BBB dysfunction as a major cerebrovascular complication in diabetes mellitus that underlies the pathogenesis of a host of CNS disorders [7,8]. Both hypoglycemia and diabetes-dependent chronic hyperglycemia have profound impact on cerebrovasculature in terms of endothelial dysfunction, increased vascular permeability, and altered gene expression, thus leading to potential neuronal injuries. Chronic hyperglycemia has been shown to affect BBB permeability, although conflicting evidence exists between animal [9,10] and human studies [11,12]. Similarly, disparate evidence from clinical and experimental studies has been reported in relation to the effects of hyperglycemia on BBB glucose transporters (mainly GLUT-1) expression and nutrient transport. While some studies have shown long-standing hyperglycemia being prodromal to a decreased GLUT-1 expression, others have reported an unaltered cerebral glucose metabolism and no changes in the expression of glucose transporter under similar conditions. Chronic hypoglycemia has been shown to elicit BBB over-expression of GLUT-1 perhaps to compensate for low circulating blood glucose levels [13]. However, the issue of whether altered glycemic conditions may impact other BBB glucose transporters has only been marginally addressed. Potential effects of altered glycemia on TJ expression/redistribution and endothelial oxidative/inflammatory responses are other under-investigated areas. Notably, inter-species variations in the expression of BBB phenotypic markers further impact the translational significance of a large number of such studies [14].

Reliable *in vitro* models that closely mimic the human BBB microenvironement are essential to understand the cellular/molecular basis of brain microvessel endothelial physiology [15], thus facilitating the identification and characterization of BBB regulatory mechanisms potentially impaired in diseased states [16,17]. Recently, an immortalized brain microvascular endothelial cell line, hCMEC/D3, was derived from isolated human primary BBB ECs by lentiviral vector-mediated co-expression of human telomerase and SV40 T antigen [18]. This stable cell line exhibits robust proliferation while retaining the morphological and known biochemical phenotype of differentiated human BBB ECs over many passages [18,19]. This cell line has been extensively characterized for its utility as a model of human BBB for CNS drug delivery and translational neurovascular research focusing on BBB function [16,20-22].

Given the increased public attention to diabetes and its relevance to the pathogenesis of major CNS disorders (e.g., Diabetic neuropathy, Alzheimer’s, Dementia, Stroke, Depression, etc.), the objective of this study is to assess and characterize the independent impact of diabetes-associated hyper and hypoglycemic conditions on BBB integrity and endothelial function using hCMEC/D3 cell line.

## Methods

### Materials and reagents

The antibodies used in this study were obtained from the following sources: Rabbit anti-ZO-1 (#D7D12) and anti-VE-cadherin (#D87F2) from Cell Signaling Technology (Danvers, MA, USA); Rabbit anti-GLUT-1 (#ab15309) and anti-SGLT-1 (#ab14686) from Abcam (Cambridge, MA, USA); Rabbit anti-GLUT-4 (#sc-7938) from Santa Cruz Biotechnology (Santa Cruz, CA, USA); Donkey anti-rabbit (#NA934) and sheep anti-mouse (#NA931) HRP-linked antibodies from GE Healthcare (Piscataway, NJ, USA); Mouse anti-Claudin 5 (#35-2500), goat anti-mouse (#A11001) and anti-rabbit (#A21428) conjugated to Alexa Fluor® 488 and 555 from Invitrogen (Camarillo, CA, USA). Sterile culture ware was obtained from Fisher Scientific (Pittsburgh, PA, USA), while other reagents and chemicals were purchased from Sigma-Aldrich (St. Louis, MO, USA) or Bio-Rad laboratories (Hercules, CA, USA). Fluorescein isothiocyanate (FITC) and Rhodamine B isothiocyanate (RITC) dextrans were purchased from Sigma-Aldrich, while Cascade Blue®-dextran was obtained from Invitrogen (Eugene, OR, USA).

### Cell culture

The immortalized hCMEC/D3 cell line was donated by Dr. Couraud (INSERM, Paris). The hCMEC/D3 cells (passage 28-32) were seeded on collagen-coated culture flasks (2.5-3 × 10^4^/cm^2^) or glass slides (4 × 10^4^/cm^2^) in EBM-2 basal medium (Lonza, Walkersville, MD, USA) supplemented with 5% FBS (Atlanta Biologicals, Lawrenceville, GA, USA), chemically defined lipid concentrate (Life technologies, Carlsbad, CA, USA), growth factors, antibiotic/antimycotic (1:1) and HEPES (10 mM) and maintained at 37°C with 5% CO_2_ exposure. Medium was changed every 2-3 days until the cells reached confluence. Monolayer integrity of hCMEC/D3 cells at confluence was confirmed by the expression of endothelial cell-specific phenotypic markers at cell-cell junctions, as previously described [18].

### Co-culture setup

HCMEC/D3 cells were co-cultured with juxtaposed human astrocytes (ScienCell Research Laboratories, San Diego, CA, USA) grown on the abluminal side of semipermeable Transwell® inserts [16,23]. The Transwell® apparatus allows for the BBB endothelium to remain in anatomical contact with astrocytes thus closely mimicking the BBB anatomical structure [17]. Briefly, Transwell® inserts (clear polyester membranes with 0.4 μm pore size) were seeded with human primary astrocytes (HA, passage no. 3-4) on the abluminal side of the microporous membrane and cultured in DMEM/F12 media with 10% FBS. After 72 h, hCMEC/D3 cells were loaded on the apical side and grown in EBM-2 basal medium containing the supplements mentioned above. Cells in co-cultures were grown in their respective media for one week before treatment. Confluence and integrity were checked by phase contrast microscopy and trans-endothelial electrical resistance (TEER) measurements.

### Treatment

HCMEC/D3 cells in monocultures or co-cultures were maintained overnight in media containing 1% human serum with no growth factors (referred as low serum media). The next day, cells were exposed to fresh low serum media containing 5.5 mM (normal/control), 2.2 mM (hypoglycemic) or 35 mM (hyperglycemic) D-glucose for 3-24 h. These concentrations were selected based on previously published reports *in vitro*[24,25] and *in vivo*[13]. Additional osmotic control experiments were performed by exposing hCMEC/D3 cells to media containing 5.5 mM D-glucose + 4.5 mM L-glucose, 2.2 mM D-glucose + 7.8 mM L-glucose or 30 mM D-mannitol. Note that this mannitol concentration was significantly lower than the 1.4 M used for transient opening of BBB [26].

### Cell viability

Following exposure to normal, hypo or hyperglycemic (5.5, 2.2 or 35 mM D-glucose respectively) media for 3 and 24 h, cell viability was determined by lactate dehydrogenase (LDH) measurements in the culture medium by a colorimetric enzymatic reaction (Pierce LDH cytotoxicity assay kit - Thermo Scientific, Rockford, IL), according to the manufacturer guidelines.

### BBB integrity

Acute effects of altered glycemia on BBB integrity were assessed by measuring concomitant paracellular permeability (luminal to abluminal) to labeled dextrans (4-70 kDa) and TEER (Ω.cm^2^) measurement by EVOM 2 (World Precision Instruments, Sarasota, FL, USA), as described earlier [16,23]. For paracellular permeability studies, hCMEC/D3 cell monolayers seeded on Transwell inserts were exposed to normal, hyper or hypoglycemic media for 30 min at 37°C in humidified incubator prior to the addition of a mixture of labeled dextrans in PBS (FITC- 4 kDa, 7 mg/ml; Cascade Blue®- 10 kDa, 5 mg/ml; and RITC - 70 kDa, 7 mg/ml) to the luminal compartment. Abluminal samples (50 μL) were collected over 60 min and replaced with equal volume of fresh media to allow sink conditions. Dextran flux was determined by fluorescent measurements at appropriate excitation and emission wavelengths for each fluorescent dye. Media-only samples with no added dextrans and cell-free inserts with added dextrans served as reference. Similarly, following 24 h treatment, TEER values were measured across hCMEC/D3 cell monolayers. Cell-free inserts were used to calculate the final resistance.

### ELISA

Following treatment, the respective conditioned media from either mono or co-cultures of hCMEC/D3 cells were analyzed by Quantikine ELISA kits (R&D systems, Minneapolis, MN, USA) for vascular endothelial growth factor (VEGF), platelet derived growth factor (PDGF-BB) and interleukin-6 (IL-6) according to the manufacturer’s protocol.

### Immunofluorescence

HCMEC/D3 cells cultured on chamber slides were rinsed in PBS and fixed with ice-cold acetone (10 min at -20°C). After PBS washes, fixed cells were blocked with 5% goat serum in PBS at room temperature (RT) for 30 min, followed by incubation with rabbit (1:200) or mouse (1:150) primary antibodies overnight at 4°C. After 3 rinses with PBS, cells were incubated for 1 h at RT with Alexa Fluor® 488 or 555 conjugated goat anti-mouse or anti-rabbit antibodies, respectively (1:1000). Thereafter, cells were rinsed and counterstained with DAPI in Prolonged Gold Anti-fade reagent (Invitrogen, OR, USA). Slides were cover slipped, cured overnight in dark and examined with EVOS digital inverted fluorescence microscope. Cell staining devoid of primary antibodies served as negative controls.

### Western blotting

Briefly, cells were lysed in ice-cold RIPA buffer containing Complete Protease Inhibitor (Roche Diagnostics, Indianapolis, IN, USA) and centrifuged at 14000 rpm, 4°C for 30 min. Protein concentration was determined by bicinchoninic acid assay [27]. Denatured samples containing equal amounts of protein (12 or 15 μg) were subjected to SDS-PAGE (7.5 or 10%) and electrotransferred to PVDF or nitrocellulose membranes. Membranes were blocked for 2 h (RT) with 5% non-fat dry milk in TBS containing 0.1% Tween-20 (TTBS) and subsequently incubated with rabbit (1:500) or mouse (1:350) primary antibodies. After 3 washes (10 min each) with TTBS, membranes were incubated with anti-rabbit or anti-mouse (1:7000) HRP-conjugated secondary antibodies (2 h, RT) and washed with TTBS. Bands were detected by enhanced chemiluminescence using Amersham ECL™ Prime with ChemiDoc™ XRS system. Membranes were subsequently stripped and probed for β-tubulin (1:500), as a loading control. Band densities were analyzed by Quantity One Software.

### Statistical analyses

Data from all experiments were expressed as mean ± standard error of mean (S.E.M) and analyzed by one or two-way ANOVA using GraphPad Prism Software Inc. (La Jolla, CA, USA). *Post hoc* multiple comparison tests were performed with Tukey’s or Dunnett’s test. P value less than 0.05 was considered statistically significant.

## Results

### Effects of altered glycemia on hCMEC/D3 cell viability

**Figure 1:**
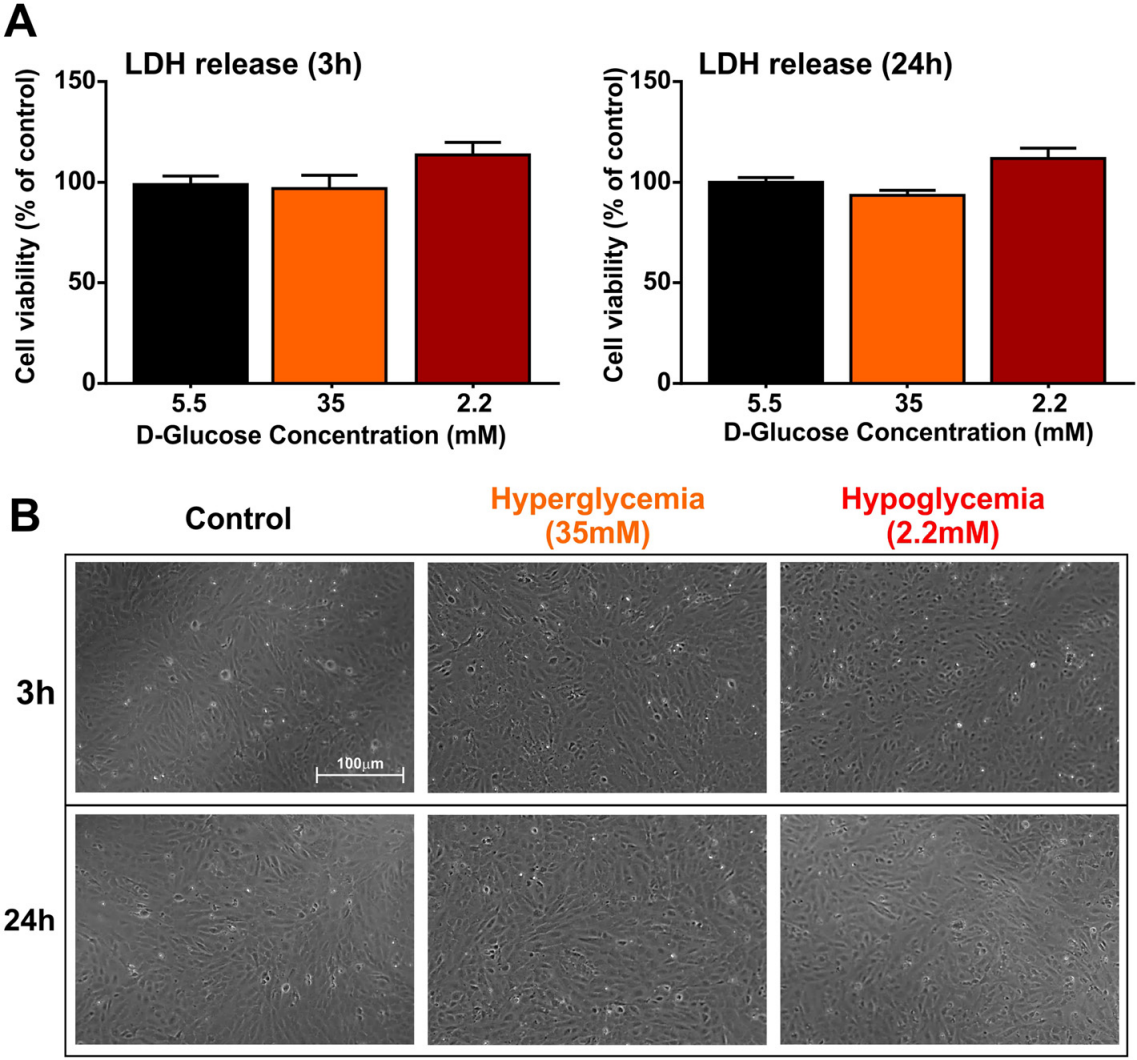
**Effects of hyper and hypoglycemia on hCMEC/D3 cell viability. (A)** LDH release (a measure of cytotoxicity) from the D3 cell monolayers at 3 and 24 h following exposure to normal, hyper and hypoglycemic conditions. Data were expressed as mean ± S.E.M. (% control). **(B)** Phase contrast microscopic images (4×, scale: 1000 μm) of the hCMEC/D3 monolayers following exposure to normal or altered glycemic conditions.

LDH release from the damaged cells (a measure of cytotoxicity) was determined to assess hCMEC/D3 cell viability at 3 and 24 h following exposure to normal, hyper and hypoglycemic media. Analysis of the results (one-way ANOVA) clearly indicate a lack of cytotoxic effect of either hypo or hyperglycemia on hCMEC/D3 under the tested exposure conditions (3 and 24 h - see Figure 1A) although hypoglycemia did induce slight (not significant) increase in LDH release at both time points when compared to controls. These data were also supported by phase contrast microscopy analysis of hCMEC/D3 cell cultures (see Figure 1B) showing complete and fully confluent cell monolayers devoid of morphological alterations and/or empty spots indicative of cell injury and death.

### Effects of hypo- and hyperglycemia on BBB TJ protein expression

**Figure 2:**
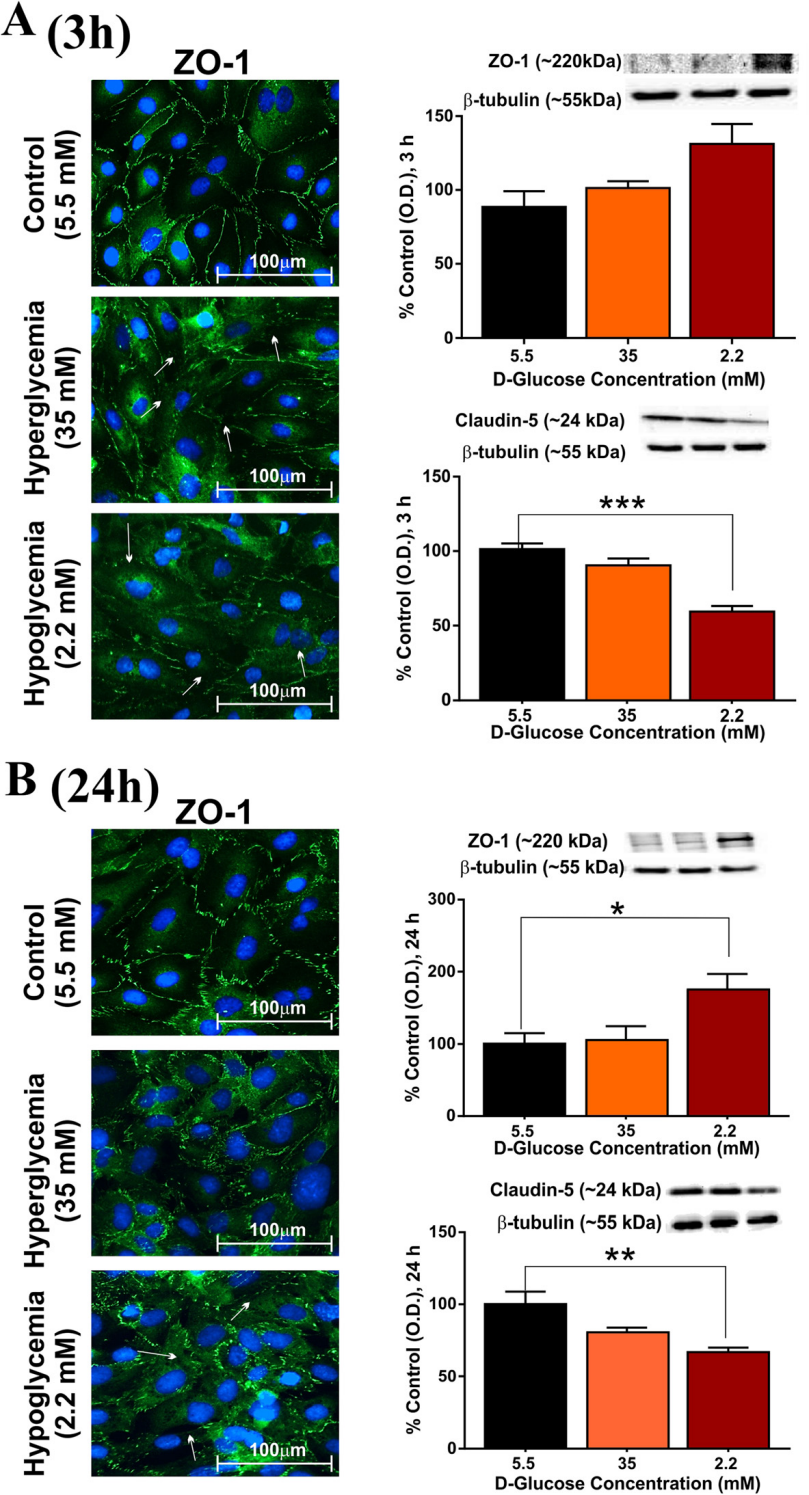
**Effects of altered glycaemia on expression and distribution of TJ proteins, ZO-1 and claudin-5.** Following exposure to control, hypo or hyperglycemia, hCMEC/D3 cells were dual-labeled for ZO-1 (green) localization by immunofluorescence analysis at 3 h **(A)** and 24 h **(B)**. Images were captured at 40× (scale: 100 μm) and merged with DAPI. Immunofluorescence images were paired with corresponding western blot analyses at 3 and 24 h. Data were expressed as mean ± S.E.M. Representative western blots were shown with β-tubulin as loading control. *** p < 0.001, ** p < 0.01, * p < 0.05 vs. control. N = 3-4 samples/condition and replicated twice.

Side-by-side comparisons of the immunofluorescence data indicate a predominant and precise distribution of membrane-associated ZO-1 along the cell-cell junctions, under normal glycemic conditions at 3 h (Figure 2A) and 24 h (Figure 2B). Exposure to hypoglycemic medium resulted in a significant disruption of the ZO-1 bands at cell-cell contacts and loss of cell junctions at 3 (Figure 2A) and 24 h (Figure 2B). This was paralleled by a significant increase in cytosolic ZO-1 expression, as supported by the western blots at 3 and 24 h (Figure 2). Hypoglycemic exposure also led to significant and sustained down-regulation of claudin-5 expression at 3 (Figure 2A) and 24 h (Figure 2B) in hCMEC/D3 cells, as demonstrated by western blots (Figure 2, p < 0.001; vs. control). In contrast to hypoglycemia, hyperglycemic exposure for 24 h did not affect the ZO-1 overall expression (Figure 2B), although disruption of ZO-1 band at cell-cell contacts (see white arrows) and a slight increase in total ZO-1 expression was observed following the initial 3 h treatment, compared to control (Figure 2A). In addition, hyperglycemia did not affect claudin-5 expression in hCMEC/D3 cell monolayers at either 3 or 24 h, as assessed by western blots (Figure 2), though a decreased level of expression (albeit not significant) was noted at 24 h exposure; Figure 2B). Importantly, these relative changes in the membrane distribution/expression of ZO-1 and claudin-5 were not related to the osmotic effects of low or high D-glucose containing media, as determined by parallel osmotic control experiments (data not shown).

### Effects of hypo- and hyperglycemia on BBB integrity

**Figure 3:**
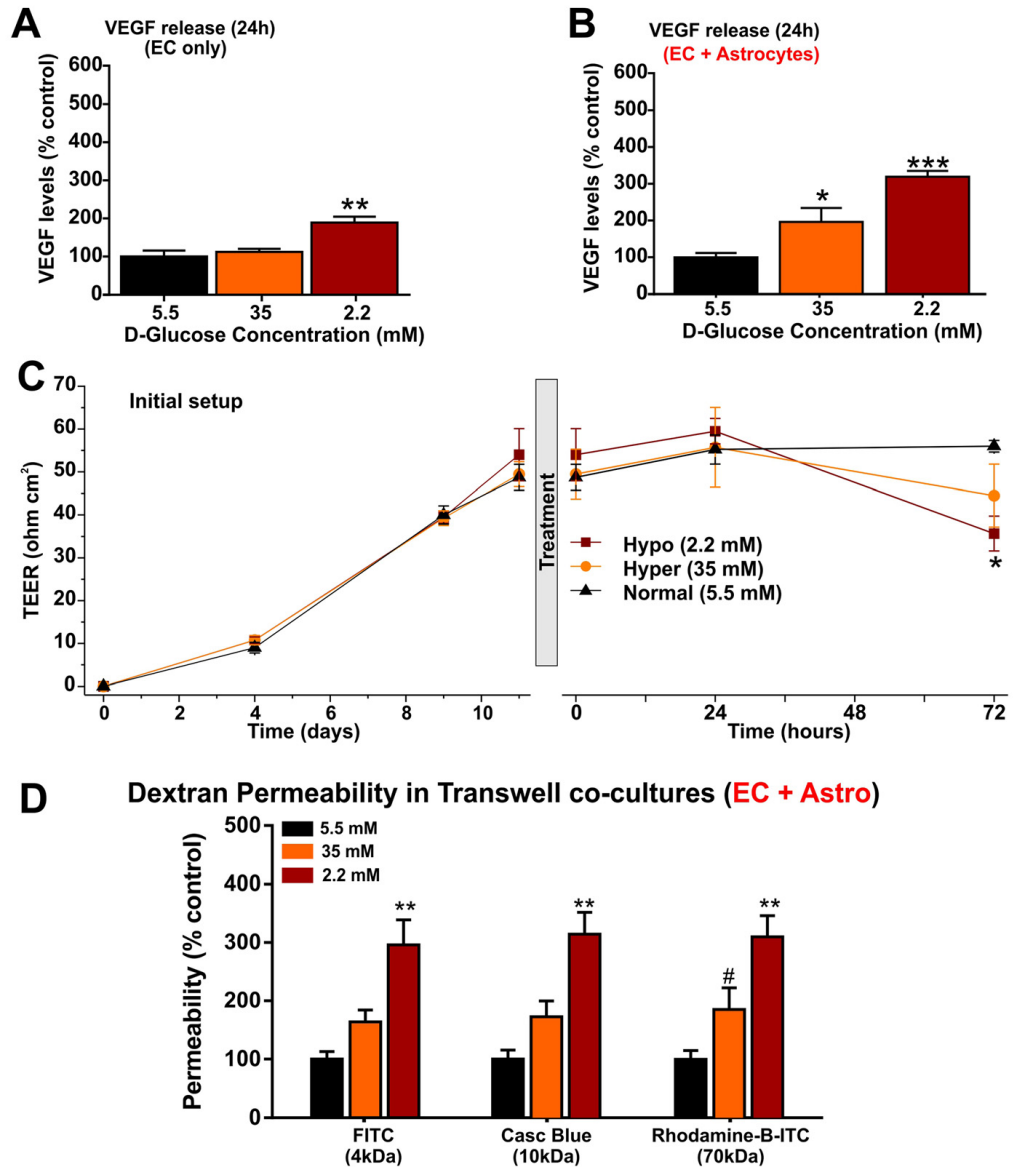
**Altered glycaemia affects BBB integrity.** Hypoglycemia induced a significant increase in extracellular VEGF release from hCMEC/D3 monocultures at 24 h **(A)**. Both, hyper and hypoglycemic exposure significantly up-regulate 24 h VEGF release from hCMEC/D3 cells co-cultured with astrocytes **(B)**. Hyper and hypoglycemia affect BBB integrity as demonstrated by TEER (ohm.cm^2^) measurement over 72 h exposure. TEER values of cell-free inserts served as a reference **(C)**. Also shown in figure were the TEER values during initial set up prior to treatment. Increase in paracellular permeability of fluorescent dextrans (4-70 kDa) expressed as percentage of control by hyper and hypoglycemia, across the monolayers of hCMEC/D3 cells (apical or luminal side) co-cultured with astrocytes (abluminal side), at 30 min following the addition of dextrans **(D)**. Briefly, hCMEC/D3 cells in Transwell inserts were exposed to altered glycemic conditions for 30 min at 37°C, prior to addition of labeled dextrans. Data were expressed as mean ± S.E.M. *** p < 0.001, ** p < 0.01, * p < 0.05 and # p < 0.05, vs. control. N = 5-6/condition and replicated twice.

VEGF, a potent mediator of vascular permeability, is known to alter BBB integrity [28]. As shown in Figure 3, one-way ANOVA revealed a significant effect of treatment on VEGF release from mono or co-cultures of hCMEC/D3 cells. Specifically, hypoglycemia induced a significant increase in VEGF release from hCMEC/D3 cells in monocultures (two-fold, p < 0.01, Figure 3A) or co-cultured with human primary astrocytes (> three-fold, p < 0.001, Figure 3B), compared to controls. Similar effect, although less pronounced, was observed under hyperglycemic conditions resulting in a significant increase in VEGF release (p < 0.05, Figure 3B). However, in contrast to hypoglycemia, 24 h of hyperglycemia did not affect VEGF release from monocultures of hCMEC/D3 cells.

On the other hand, TEER values measured across the hCMEC/D3 monolayers remained relatively stable and were not affected following an initial 24 h exposure to hypo or hyperglycemia compared to controls, as shown in Figure 3C. However, a significant TEER decrease was observed following 72 h hypoglycemic exposure (vs. control, p < 0.05). TEER was only moderately decreased by hyperglycemia over the same time of exposure.

Consistent with VEGF release, BBB permeability to fluorescent dextrans was also affected. HCMEC/D3 cells in co-cultures were exposed to normal, hyper or hypoglycemic conditions for 30 min at 37°C and then added a mixture of labeled dextrans (see Methods). Permeability was measured in acute over 30 min time window following the addition of dextrans. As shown in Figure 3D, paracellular permeability/abluminal accumulation (μg/ml) of all labeled dextrans (expressed as% controls: 0.195 ± 0.007 × 10^-3^ cm/min; 0.0975 ± 0.005 × 10^-3^ cm/min and 0.008 ± 0.0004 × 10^-3^ cm/min for 4, 10 and 70 kDa respectively) was significantly enhanced by hypoglycemia over time at 30 min (following the addition of dextrans). Specifically, the permeability to 4- and 10 kDa dextrans increased within 15 min (data not shown) and was significant at 30 min (p < 0.01 or p < 0.05 vs. control), while the paracellular flux of 70 kDa dextran reached statistical significance only at 30 min following the addition of dextrans to the lumenal side (p < 0.001, vs. control). These results strongly suggest that even short exposure to severe hypoglycemic conditions can affect the integrity and stringency of the BBB. Similarly, acute hyperglycemia progressively and significantly increased the permeability of 4- and 10 kDa dextrans (including 70 kDa) by 30 min (p < 0.05, vs. control) following the addition of dextrans. Importantly, the alteration in BBB integrity measured by the abluminal accumulation of dextrans was not related to the osmotic effects of abnormal D-glucose concentrations in the media. For example, hypoglycemic media containing 7.8 mM L-glucose showed a similar increase in dextran permeability, compared to an osmotically equivalent normoglycemic medium containing 4.5 mM L-glucose (data not shown).

### Hypoglycemia affects expression of glucose transporters in BBB endothelial cells

**Figure 4:**
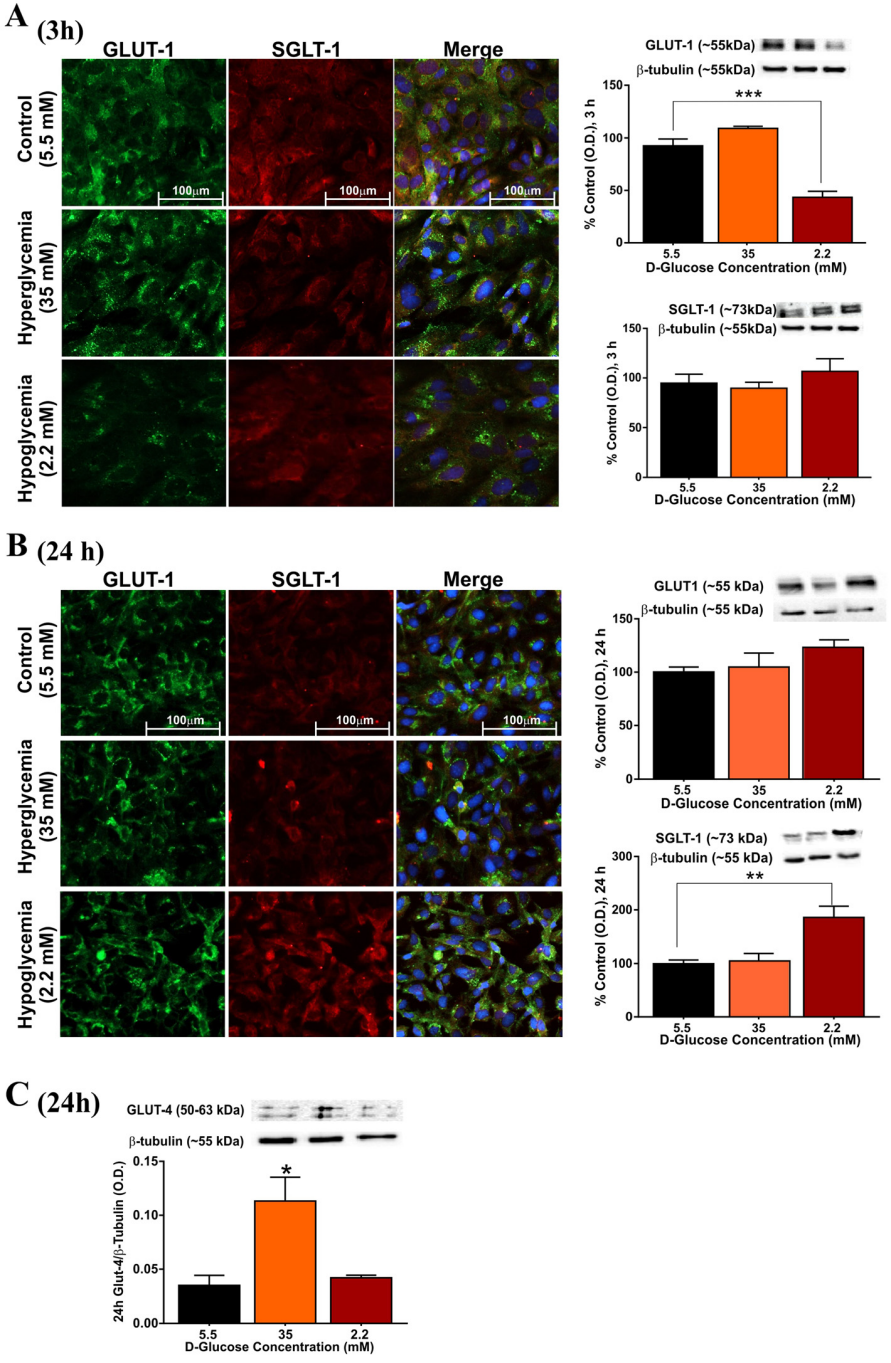
**Effects of hyper and hypoglycemia on the expression and distribution of glucose transporters.** Following exposure to control or altered glycemic conditions, hCMEC/D3 cells were co-stained for immunofluorescence analysis of GLUT-1 (green) and SGLT-1 (red) expression and distribution at 3 h **(A)** and 24 h **(B)**. Images were captured at 40× (scale: 100 μm) and merged with DAPI. Results were corroborated by western blot analyses at 3 and 24 h. Expression of GLUT-4 was assessed by western blot analysis at 24 h following altered glycemia **(C)**. Data were expressed as mean ± S.E.M. Representative western blots were shown with β-tubulin as loading control. *** p < 0.001, ** p < 0.01, * p < 0.05 and # p < 0.05, vs. control. N = 3-4 samples/condition and replicated twice.

Immunofluorescence analysis of hCMEC/D3 cultures exposed to hypoglycemia for 3 h revealed a significant decrease in the expression of GLUT-1 which was corroborated by western blots as shown in Figure 4A (p < 0.001, vs. control). Note however, that the effect was transient as the GLUT-1 expression returned to the levels comparable or slightly higher than control at 24 h (Figure 4B). No changes in GLUT-1 expression were noted in cultures exposed to hyperglycemic conditions. By contrast, 24 h hypoglycemia significantly increased the expression level of the SGLT-1, a sodium-dependent glucose transporter, as clearly shown by immunofluorescence analysis of hCMEC/D3 monolayers and corresponding western blots (see Figure 4B). However, no significant changes were observed at 3 h hypoglycemia. Importantly, neither 3 h nor 24 h hyperglycemia elicited any expression change of SGLT-1, when compared to controls (Figure 4A & B; p < 0.01, vs. control). Interestingly, the expression level of GLUT-4 was up-regulated by hyperglycemia, but remained unchanged under hypoglycemic culture conditions (Figure 4C; p < 0.05). Importantly, these relative changes in glucose transporter expression were not related to the osmotic effects of low or high D-glucose containing media, as determined by parallel osmotic control experiments mentioned above (data not shown).

### Effects of hyper and hypoglycemia on BBB endothelial cell oxidative and inflammatory stress pathways

**Figure 5:**
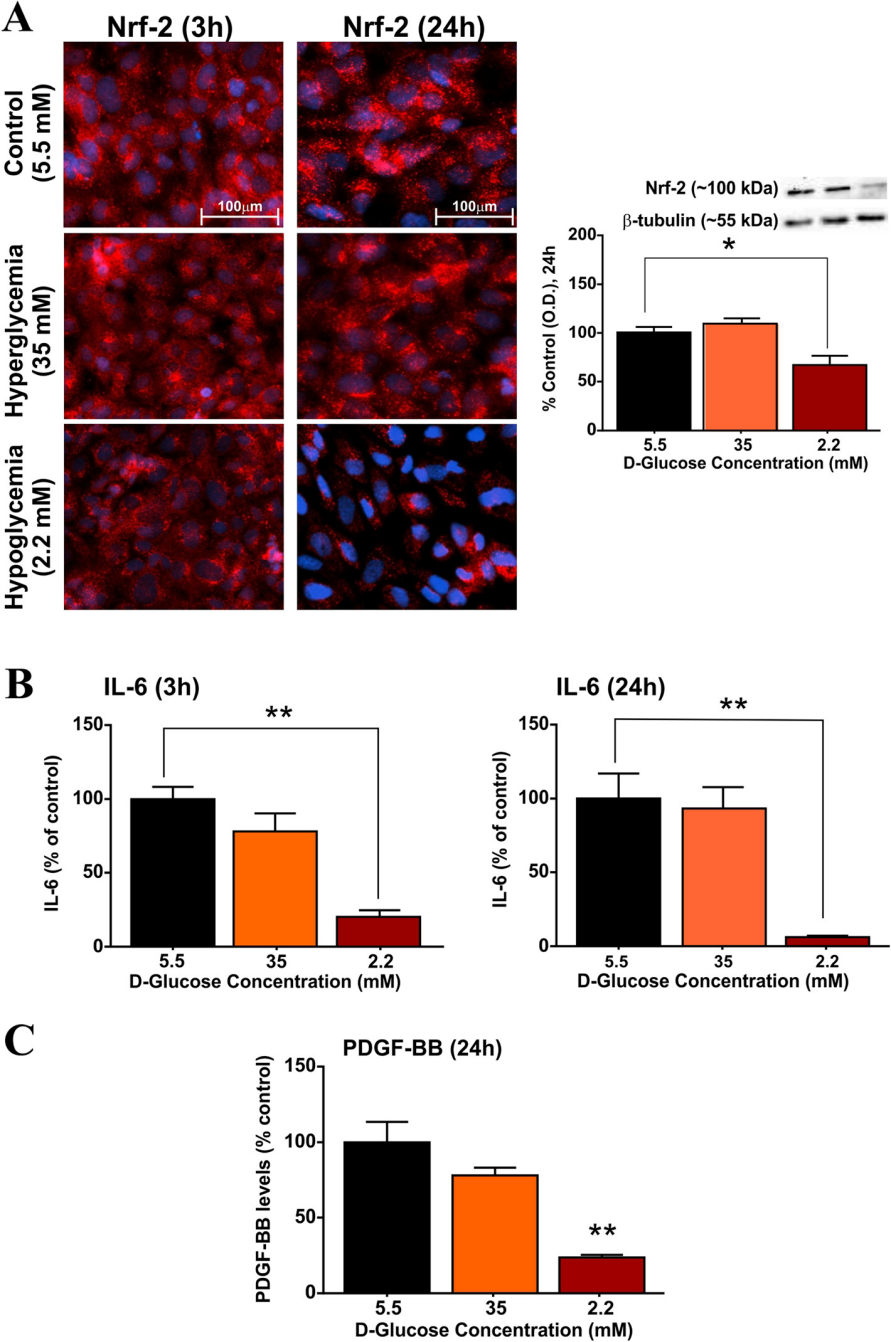
**Effects of altered glycaemia on oxidative stress pathways in BBB endothelial cells.** Briefly, hCMEC/D3 cells were exposed to normal, hyper and hypoglycemic conditions for 3-24 h. **(A)** The expression and distribution of Nrf-2 (transcription factor involved in the cell’s anti-oxidative defense) was assessed by immunofluorescence and western blot analysis at 3 and 24 h. Images were captured at 40× (scale: 100 μm) and merged with DAPI. **(B)** Endothelial release of IL-6 following treatment at 3 and 24 h was quantified by ELISA. Data were expressed as mean ± S.E.M. (% control). β-tubulin was used as loading control. N = 3-5 samples/condition and replicated twice. Hypoglycemia down-regulates the endothelial release of PDGF-BB **(C)**.

Nuclear-factor (erythroid derived 2) related factor-2 (Nrf2) is a potential transcription factor involved in the cell’s endogenous defense against oxidative stress by inducing the transcription of cytoprotective anti-oxidant genes [29,30]. Immunofluorescence data indicate a progressive down-regulation of the cytosolic expression of Nrf-2 in hCMEC/D3 cells exposed to hypoglycemic culture conditions which became statistically relevant at 24 h. This finding was supported by corresponding western blot data (Figure 5A; p < 0.05 vs control). Further, as shown in Figure 5B, hypoglycemia significantly decreased the release of interleukin-6 (IL-6, a pro-inflammatory cytokine that holds a key role in the inflammatory signaling at BBB [31]), from hCMEC/D3 monolayers at 3 h (~4-fold decrease, p < 0.01, vs. control) and 24 h (~10-fold decrease, p < 0.01, vs. control). In contrast to hypoglycemia, 24 h hyperglycemic exposure did not alter total cellular Nrf-2 expression total or the endothelial release of IL6, at either 3 or 24 h exposure, as compared to controls (Figure 5B). Release of IL-1β was unaffected by either conditions (data not shown).

As shown in Figure 5C, exposure to hypoglycemic media significantly decreased the endothelial release of PDGF-BB at 24 h (p < 0.01, vs. control). Although, slight decrease in the PDGF-BB release from hCMEC/D3 cells was noticed following exposure to hyperglycemic conditions, it failed to reach statistical significance (Figure 5C).

### Altered glycemia affects the distribution and expression of VE-cadherin and vascular cell adhesion molecule-1 (VCAM-1) in BBB endothelial cells

**Figure 6:**
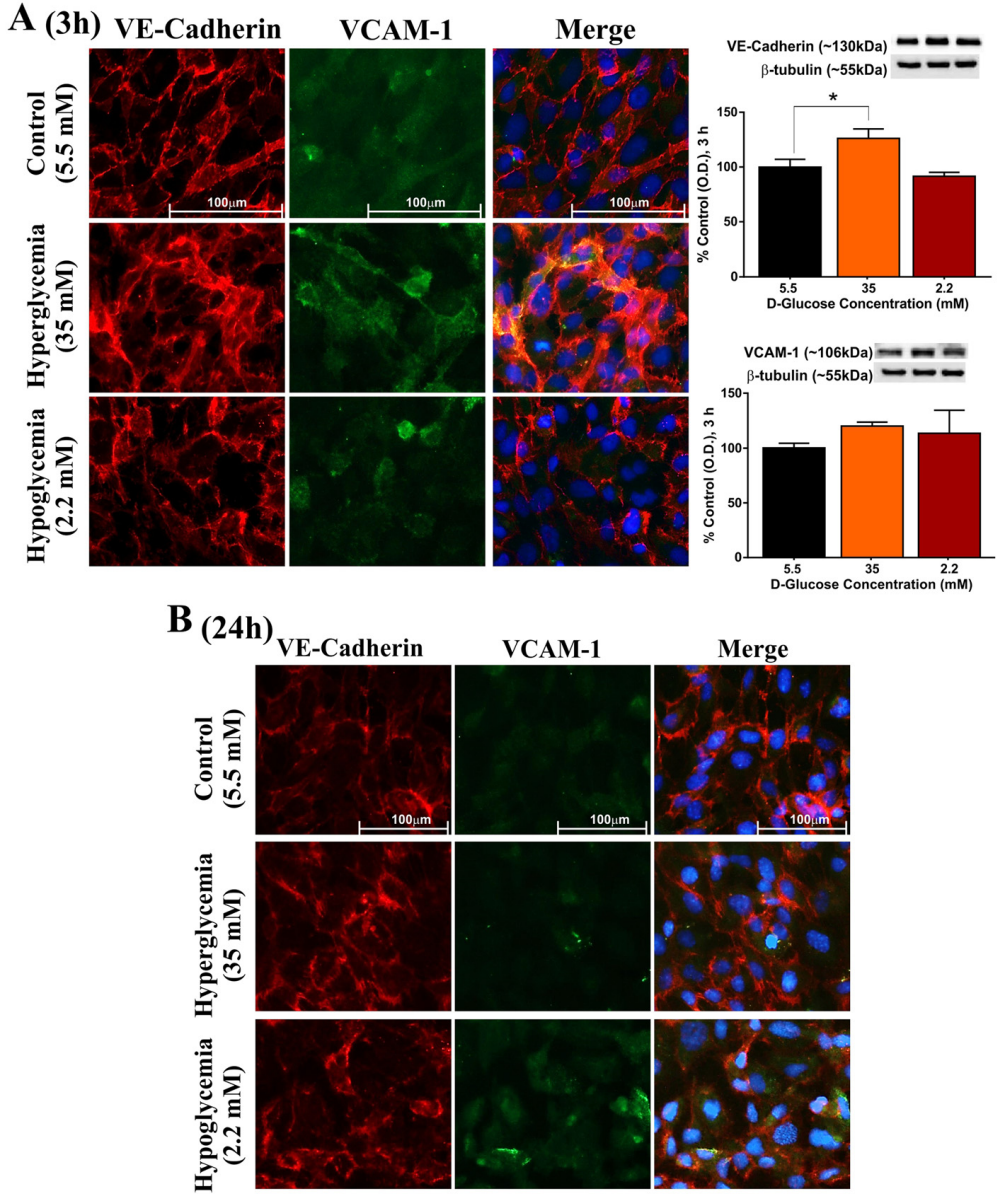
**Altered glycaemia-induced increase in BBB endothelial cell inflammation.** Following exposure to normal or altered glycemic conditions for 3 h **(A)** and 24 h **(B)**, hCMEC/D3 cells were co-stained for the expression and distribution of VE-cadherin (red) and VCAM (green), important modulators of inflammation. Images were captured at 40× (scale: 100 μm) and merged with DAPI. Representative western blots were shown with β-tubulin as loading control. Data were expressed as mean ± S.E.M. (% control). N = 3-4 samples/condition and replicated twice.

As shown in Figure 6, immunofluorescence analyses at 3 and 24 h suggest disruption of membrane-associated VE-cadherin (an endothelial calcium-dependent adhesion glycoprotein located at cell-cell contacts which regulates BBB integrity [3,19,32]), following hypoglycemic exposure for 3 and 24 h. This effect was accompanied by a cytosolic redistribution of the protein when compared to controls. Note that western blots at 3 h exposure did not show any significant change in total VE-cadherin expression (Figure 6A). Similar effects on VE-cadherin distribution (disruption of VE-cadherin at cell-cell contacts) was observed under hyperglycemic condition, although in contrast to hypoglycemia, hyperglycemia significantly increased total VE-cadherin expression, as demonstrated by western blots (p < 0.05, vs. control, Figure 6A) at 3 h. However, at 24 h, alterations of VE-cadherin distribution/expression (Figure 6B) were not observed, thereby suggesting the effect was transient.

In addition to the disruption of VE cadherin, a modest (not statistically significant) increase of VCAM-1 (both membrane and soluble fractions) was observed following 3 and 24 h exposure to hypoglycemia (Figure 6A & B). However, VCAM-1 expression changes observed under hyperglycemic conditions were transient (Figure 6A & B).

## Discussion

*In vivo* experiments are currently the gold standard in basic and translational studies; however several factors generally hinder the utility of animal models to dissect out the impact of complex pathophysiological stimuli down to molecular mechanisms. In addition, marked inter-species differences make it difficult to extrapolate *in vivo* data to humans. In this context, development of reliable *in vitro* platforms for basic and translational study is supported by the National Research Council [33]. Recent studies have shown inter-species variations related to the expression and distribution of BBB transporters, thus advocating for the use of humanized models to study *in vitro* the physiological/pathological responses of the human BBB endothelium *in vivo*[14,17,34]. Although, various immortalized endothelial cell lines have been developed, the hCMEC/D3 cell line stands out as the most well characterized *in vitro* human BBB model till date [20]. It has been shown to retain known structural and functional aspects of differentiated human BBB endothelium [16,20] over multiple passages [18,20]. However, the expression of specific TJ molecules and transporters on hCMEC/D3 cells, though comparable to primary BMECs [18,35], remained relatively 3-5 fold lower than in isolated human brain microvessels [36]. Recently, Luissint and colleagues [37] have investigated the molecular partners of claudin-5 and their putative involvement in regulation and maintenance of BBB TJ integrity in this cell line. Nevertheless, hCMEC/D3 endothelial cells maintain a restrictive barrier with expression of functional transporters and intercellular TJs that can be further enhanced by various stimuli and culture conditions including exposure to astrocytes and shear stress.

Both claudin-5 and ZO-1 have previously been shown to play a critical role in BBB TJ formation and organization [16,38,39]. Our data demonstrate a progressive down-regulation of claudin-5 expression at cell-cell contacts following hypoglycemic exposure, while exposure to hyperglycemia did not alter its immunoreactivity, in accordance with previous reports [24]. Surprisingly, an increased expression of cytosolic ZO-1 was observed at 3 and 24 h under hypoglycemic conditions. However, prolonged exposure (72 h, data not shown) resulted in significant reduction of ZO-1, suggesting a time-dependent dysregulation of ZO-1 at cell-cell contacts by hypoglycemia. It is plausibly explained that hypoglycemic exposure for 3-24 h induces a significant translocation of ZO-1 away from membrane, thus affecting the barrier integrity, as discussed below. On the other hand, hyperglycemia for 24 h did not alter ZO-1 expression, as reported earlier [40], although a significant alteration in its localization (a pronounced cytosolic distribution) was observed.

VEGF - an endothelial specific mitogen and a potent mediator of vascular permeability [28], was shown to alter BBB integrity *in vitro*[41] and *in vivo*[38] by affecting TJ protein expression and organization including phosphorylation. In addition, our findings are also supported by previous studies performed in brain [24,41] and retinal ECs [42]. Notably, our results have shown significant differences in endothelial response to hypo or hyperglycemic conditions which manifested through differential release of VEGF when ECs were cultured with or without abluminal astrocytes (Figure 3). Specifically, VEGF release in response to hypoglycemic media was significantly enhanced when ECs were co-cultured with astrocytes as compared to endothelial monocultures. Interestingly, hyperglycemic exposure showed a two-fold increase in VEGF release by endothelial cells co-cultured with astrocytes (Figure 3B), while no significant effects were observed in monocultures when compared to controls (Figure 3A). However, while no significant TEER changes were observed within 24 h treatment (despite the initial increase of VEGF), we observed a significant increase in permeability to dextran molecules. A statistically significant TEER decrease was observed only at 72 h which was paralleled by a larger increase in VEGF release (data not shown) compared to both controls and 24 h exposure (Figure 3C). While we might have expected a decline in TEER even at 24 h this is not surprising since characteristically relatively low TEER values commonly obtained (with few exceptions) in Transwell setups may not provide enough sensitivity to detect minimal yet significant changes in paracellular permeability. Such is the case here where relative loss of BBB integrity was instead demonstrated by changes in dextran permeability. Nevertheless, hypo and hyperglycemia differentially regulate the TJ protein distribution and expression in a time-dependent manner and thus negatively affect the BBB integrity and corresponding selective permeability. While the mechanism(s) for the observed effects at this point are still unknown it is possible that the increase in VEGF expression can be responsible for these phenomena although other mechanisms (e.g. oxidative/inflammatory stress as mentioned below) cannot be ruled out and need to be further investigated.

In line with these findings, our results demonstrate that hypoglycemia, and hyperglycemia (to some extent), potentially compromise BBB integrity, as evidenced by a significant increase in paracellular permeability (abluminal accumulation) to dextrans in a time-dependent and size-selective manner in hCMEC/D3 cells co-cultured with astrocytes, with a significant increase in permeability to all labeled dextrans at 60 min following treatment (vs. controls, Figure 3C). These results are supported by a recent finding in which rapid normalization of plasma glucose in diabetic rats with acute insulin injection (mimicking acute hypoglycemia) increased the sucrose permeability at 20 min [43]. Note that similar to sucrose, dextrans cross the BBB mainly by a paracellular pathway, but not through cell transcytosis [44]. Moreover, hypoglycemia induced a similar increase in paracellular flux of all labeled dextrans at 60 min (data not shown). However, a closer analysis of permeability at later time points indicates that the paracellular fluxes of all dextrans inclined towards steady state dynamics and therefore, permeability was assessed at 30 min following the addition of dextran markers.

Brain microvascular endothelium is highly enriched with stereospecific glucose transporters, mainly GLUT-1 (~55 kDa), that selectively facilitate the diffusion of glucose to the CNS [45]. Existing evidence indicates a decreased GLUT-1 activity and glucose clearance in chronic hyperglycemia, with a parallel decrease in expression of GLUT-1 at BBB [46], as confirmed by subsequent studies [47,48]. On the other hand, chronic hypoglycemia increased BBB glucose transport resulting from a concomitant increase in GLUT-1 transcription and expression on lumenal side of brain capillaries [49]. In line with these observations, we found opposite effects of hypo and hyperglycemia on GLUT-1 expression on hCEMC/D3 cells following 3-24 h exposure. Specifically, GLUT-1 expression was progressively up-regulated by hypoglycemia (24-72 h), following an initial down-regulation at 3 h. Given the differential regulation of GLUT-1 expression by hypoglycemia in a time-sensitive manner, additional studies investigating the effects of hypoglycemia on glucose transport kinetics would be important, as GLUT-1 impairment was previously linked to loss of BBB integrity [50]. Our results also indicate a marginal decrease in membrane GLUT-1 expression by prolonged hyperglycemia (slow onset). Interestingly, 24 h hyperglycemia (but not hypoglycemia) significantly increased the expression of insulin-responsive GLUT-4. Divergent evidence has been reported on the expression of this insulin-sensitive glucose transporter across various brain regions and the cerebral vasculature. However, GLUT-4 was shown to be expressed in fore brain microvessel endothelium [51], while Ngarmukos and colleagues reported its expression and co-localization with GLUT-1 and other endothelial marker (such as ZO-1) in brain glucose-sensing region [52] and could be involved in glucose uptake in hCMEC/D3 cells [53]. Nevertheless, the functional relevance of this finding is not well understood given the evidence that GLUT-4 expression at brain microvasculature remains stable during long-standing diabetes [51].

Importantly, we show for the first time, the differential regulation of BBB SGLT-1 (~75 kDa isoform) expression by hypo and hyperglycemia in hCMEC/D3 cells. Data indicate an induction of this sodium-dependent glucose transporter (2Na^+^/Glucose) expression specifically by hypoglycemic exposure from 3-24 h. These findings corroborate previous studies demonstrating an increased activity of SGLT-1 in bovine brain artery ECs during low glucose conditions [54], in addition to its role at cerebrovasculature during oxygen-glucose deprivation [55]. Overall, the present study highlights the concomitant alterations in BBB endothelial expression of GLUT-1 and SGLT-1 under hypoglycemic conditions and further studies are required to dissect out the relative functional significance of each transporter.

Existing evidence implicates the potential role of oxidative and inflammatory stress, through overproduction of reactive oxygen species (ROS), in the development of various CNS complications in diabetes [56-58]. In fact, the cerebrovascular endothelium is highly vulnerable to the oxidative stress resulting in the loss of BBB function and integrity, *via* altered composition of the intercellular TJ complexes as one of the mechanisms [59-61]. Recent studies have demonstrated the potential role of Nrf-2 in preventing the BBB dysfunction and preserving its integrity, by protecting the BBB endothelium from oxidative and inflammatory stress [30,62,63]. Here in, we demonstrate for the first time a progressive and significant loss of Nrf-2 expression by hypoglycemic insult at 24 h suggesting loss of potential protective mechanisms against oxidative stress thereby leading to increased production of ROS as previously reported by others [25] (Figure 5A), although, it cannot be ruled out that the observed decrease in Nrf-2 can be related to decrease in cellular metabolism following hypoglycemia. It is logical to assume that hypoglycemia-induced dysregulation of TJ proteins is possibly mediated through the down-regulation of Nrf-2 expression, as Nrf-2 activation was shown to partially restore TJ proteins and prevent BBB permeability [63]. In contrast, hyperglycemic exposure for 24 h did not affect Nrf-2 expression (relative to control), while previous studies indicate Nrf-2 down-regulation following chronic hyperglycemic exposure over 4 days [64].

Interestingly, we observed a significant decline in BBB endothelial IL-6 release following 3 and 24 h exposure to hypoglycemic (but not hyperglycemic) conditions (Figure 5B) which could be attributed to the down-regulation of Nrf-2. This notion is supported by a recent finding from Wruck and colleagues [65] that demonstrates the role of Nrf-2 in regulation of IL-6 gene transcription and suggests a possible role for IL-6 in oxidative stress defense. Moreover, our data indicate disruption and relocation of VE-cadherin with a concomitant increase in the soluble VCAM-1 expression, by hypo and hyperglycemia in hCMEC/D3 cells, effects that are critical to progression of BBB inflammation and augmented leukocyte infiltration in diabetes [31,59,66,67]. Interestingly, VE-cadherin was shown to positively regulate the transcription of claudin-5 [32], thus providing an alternative explanation for loss of claudin-5 by hypoglycemia. In addition, hypoglycemic exposure was shown to down-regulate the PDGF-BB release from hCMEC/D3 cells (Figure 5C). While the role of PDGF-BB in BBB physiology and function is emerging, existing evidence indicates the importance of PDGF-BB/PDGFR-β signaling for optimal function of pericytes [68], an inherent constituent of neurovascular unit that regulates the BBB function [6]. Taken together, these results suggest that immunological function of BBB is compromised following altered glycaemia and, thus may lead to subsequent neuroinflammation.

In summary, our in vitro study suggests that hypo and hyperglycemia differentially impact and potentially impair BBB integrity and function through altered expression/distribution of TJ proteins including various glucose transporters. In addition, hypoglycemic exposure affected the BBB endothelial expression of oxidative and inflammatory stress markers including VCAM-1 and Nrf-2. In conclusion, this study provides further insights into the role and modality of hypoglycemia-induced BBB dysfunction.

## Abbreviations

ANOVA: Analysis of variance; BBB: Blood-brain barrier; CNS: Central nervous system; EC: Endothelial cell; ELISA: Enzyme linked immunosorbent assay; EMB: Endothelial basal medium; FBS: Fetal bovine serum; FITC: Fluorescein isothiocyanate; GLUT-1: Glucose transporter-1; GLUT-4: Glucose transporter-4; hCMEC/D3: Immortalized human cerebro microvascular endothelial cell/D3; HRP: Horse radish peroxidase; LDH: Lactate dehydrogenase; IL-6: Interleukin-6; Nrf-2: Nuclear-factor (erythroid derived 2) related factor-2; PAGE: Polyacrylamide gel electrophoresis; PBS: Phosphate buffered saline; PDGF-BB: Platelet derived growth factor-BB; RIPA: Radio-immunoprecipitation assay; RITC: Rhodamine B isothiocyanate; ROS: Reactive oxygen species; SDS: Sodium dodecyl sulfate; SGLT-1: Sodium dependent glucose transporter-1; TBS: Tris buffered saline; TEER: Trans-endothelial electrical resistance; TJ: Tight junction; VCAM-1: Vascular cell adhesion molecule-1; VEGF: Vascular endothelial growth factor; ZO-1: Zonula Occludens-1.

## Competing interests

No competing interests are perceived to exist for any of the authors listed on this manuscript.

## Authors’ contributions

RS contributed to the experimental design, conducted the experiments and had written the manuscript; SP contributed to design and performing the experiments including the revision of the manuscript; LC contributed to experimental design, manuscript writing and revision as well as supporting this study. All authors have read and approved the final version of the manuscript.
